# Osteogenesis Imperfecta: characterization of fractures during pregnancy and post-partum

**DOI:** 10.1186/s13023-021-02148-x

**Published:** 2022-01-28

**Authors:** Eugénie Koumakis, Valérie Cormier-Daire, Azeddine Dellal, Marc Debernardi, Bernard Cortet, Françoise Debiais, Rose-Marie Javier, Thierry Thomas, Nadia Mehsen-Cetre, Martine Cohen-Solal, Elisabeth Fontanges, Michel Laroche, Valérie Porquet-Bordes, Christian Marcelli, Alexandra Benachi, Karine Briot, Christian Roux, Catherine Cormier

**Affiliations:** 1 Rheumatology Department, Cochin Hospital, Paris, AP-HP Centre-Paris University, Reference Center for Rare Genetic Bone Disorders-Cochin-constitutive site, Cochin Hospital, 27 Rue du Faubourg Saint-Jacques, 75014 Paris, France; 2 Clinical Genetics, Reference Center for bone disorders, INSERM UMR 1163, Imagine Institute, Necker Enfants-Malades Hospital, AP-HP, Paris University, Paris, France; 3 Department of Rheumatology and ULR 4490 (MABLAB), Competence Center for Rare Genetic Bone Disorders, University-Hospital of Lille, 59000 Lille, France; 4 Department of Rheumatology, CHU Poitiers; CNRS ERL7003, University of Poitiers, Poitiers, France; 5 Rheumatology Department, Competence Center for Rare Genetic Bone Disorders, University-Hospital of Strasbourg, 67098 Strasbourg, France; 6 Department of Rheumatology, CHU Saint-Etienne, INSERM U1059, Université de Lyon, Saint-Etienne, France; 7 Service de Rhumatologie, Centre de Compétence MOC et Dysplasie Fibreuse, CHU Bordeaux-Tripode, Bordeaux, France; 8 Biocar Inserm U1132 and Université de Paris, Hôpital Lariboisière, 75010 Paris, France; 9 Department of Rheumatology, Hôpital Edouard Herriot, CHU de Lyon, Lyon, France; 10 Centre de Rhumatologie, CHU Purpan, 1 place du Dr Baylac, 31059 Toulouse Cedex, France; 11 Endocrine, Bone Diseases, and Genetics Unit, Reference Centre for Rare Diseases of the Calcium and Phosphate Metabolism, ERN BOND, OSCAR Network, Children’s Hospital, Toulouse University Hospital, Toulouse, France; 12 Department of Rheumatology, CHU Caen, Caen, France; 13 Departement of Obstetrics, Gynecology and Reproductive Medicine, Hôpital Antoine-Béclère – Hôpitaux Universitaires Paris-Sud, Le Kremlin-Bicêtre, France; 14 INSERM UMR 1153, INSERM, PRESS Sorbonne Paris-Cité, Paris, France

**Keywords:** Osteogenesis Imperfecta, Pregnancy, Breastfeeding, Fracture

## Abstract

**Background:**

Pregnancy and breastfeeding are associated with bone density loss. Fracture occurrence during pregnancy and post-partum, and its determinants, remain poorly known in Osteogenesis Imperfecta (OI). The aim of this study was to characterize fractures that occurred during pregnancy and post-partum in OI patients.

**Results:**

We conducted a retrospective multicentric study including a total of 50 previously pregnant OI women from 10 Bone Centers in France. Among these patients, 12 (24%) patients experienced fractures during pregnancy or in the 6 months following delivery, and 38 (76%) did not experience any fracture. The most frequent localizations were: proximal femur (25%), spine (25%), distal femur (12.5%), and pelvis (12.5%). Fractures during pregnancy occurred during the third trimester and post-partum fractures occurred with a mean delay of 2 months following delivery. No fractures occurred during childbirth.

We next compared the 12 patients with pregnancy or post-partum fractures with the 38 patients without fractures. Mean age at pregnancy was 32.7 ± 3.1 years-old in the fractured group, vs 29.3 ± 5.0 years-old in the non-fractured group (*p* = 0.002). Breastfeeding was reported in 85.7% of patients in the fractured group, vs 47.1% in the non-fractured group (*p* = 0.03). All patients with post-partum fractures were breastfeeding. Bone mineral density was significantly lower in patients with pregnancy-related fractures compared with other patients: spine Z-score − 2.9 ± 1.6DS vs − 1.5 ± 1.7DS (*p* = 0.03), and total hip Z-score − 2.0 ± 0.7DS vs − 0.5 ± 1.4DS (*p* = 0.04). At least one osteoporosis-inducing risk factor or disease other than OI was identified in 81.8% vs 58.6% of fractured vs non-fractured patients (not significant). Fracture during pregnancy or post-partum was not associated with the severity of OI. Bisphosphonates before pregnancy were reported in 16.7% and 21.1% of patients with pregnancy-related fractures and non-fractured patients, respectively (not significant).

**Conclusions:**

OI management during pregnancy and post-partum should aim for optimal control of modifiable osteoporosis risk factors, particularly in patients with low BMD. Breastfeeding should be avoided.

## Introduction

Osteogenesis Imperfecta (OI) is a primary bone fragility disorder with an estimated prevalence of 1/15,000 births. The majority of OI cases (85–90%) is inherited in an autosomal-dominant manner and is mostly caused by mutations in *COL1A1* and *COL1A2* encoding type I collagen subunits, a major protein of the bone extracellular matrix [1, 2]. “A number of other genes have been identified more recently and the majority of them encode proteins involved in post-translational modifications of type I collagen. Among them, *IFITM5* is responsible for a rare form of dominant OI with hyperplastic callus (5%) [3]. Other recessive, dominant or X-linked forms of OI are rare (5%, 22 genes, reviewed in Marini et al. [4] and Mortier et al. [5]) and no molecular basis is found in approximately 10% of OI cases. The resulting phenotype is traditionally classified into five OI types (I to V) depending on the severity of bone fragility. The spectrum of severity of the disease is very wide, ranging from mild to moderate phenotypes (I and IV) sometimes presenting a diagnostic challenge in adults with late onset, to severe bone deformities, mobility impairment and perinatal lethality [6]. The main features of the disease are low bone mineral density (BMD) and increased bone fragility, resulting in multiple fractures occurring for minor trauma [7, 8]. Other OI clinical features may also involve extra skeletal tissues and organs, such as blue sclera, dentinogenesis imperfecta and post-pubertal hearing loss [5].

As a result of improved paediatric healthcare of OI patients, life expectancy of individuals with OI has increased beyond childhood [9]. Adults with OI now face classic life milestones, such as pregnancy and motherhood.

Pregnancy and breastfeeding are physiological conditions associated with bone metabolism alterations. Indeed, even in women without bone fragility, BMD assessment before and after pregnancy demonstrates BMD decrease during pregnancy [10]. Breastfeeding is also characterized by marked and temporary decreases in BMD, with restoration of bone density occurring within 6 to 12 months after weaning [11, 12]. Trabecular bone loss may reach up to 10% during lactation [13].

This physiological and transient bone loss is not associated with increased risk of fractures in normal conditions. Instead, women who experience fractures during pregnancy and lactation are more likely to have additional risk of fragility fractures [14].

In OI, fracture occurrence during pregnancy and post-partum, and the determinants of these fractures, are not well known. Very scarce data from a limited number of studies are available. These studies focused on obstetrical outcomes in OI patients, and were generally based on self-reports of fracture or back pain occurring around time of delivery [15–17]. Moreover, there is currently no data regarding post-partum fractures in OI.

Therefore, the aim of this study was to characterize fractures that occurred during pregnancy and post-partum in a cohort of women with OI.

## Patients and methods

### Study population

**Fig. 1:**
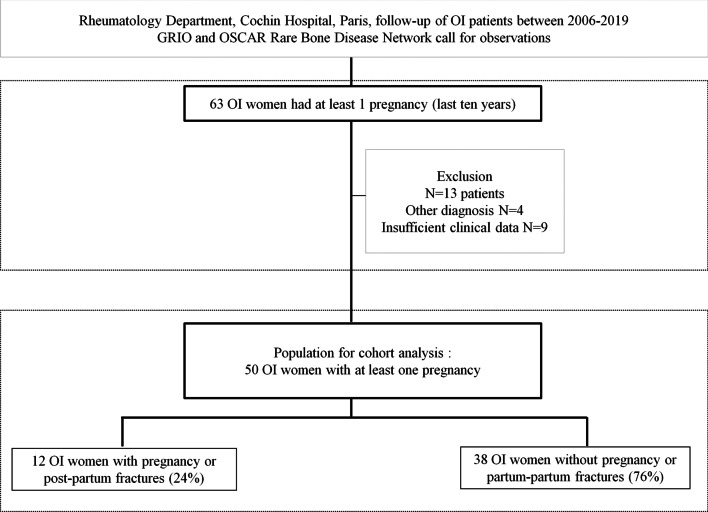
Flow-chart of patient inclusion in this female OI cohort

We performed a retrospective cohort study including women with a clinical diagnosis of OI assessed in Rheumatology department, Cochin Hospital, between January 2006 and December 2019. We also included women from 9 other Rheumatology Departments in France with expertise in bone diseases (Lille, Poitiers, Strasbourg, Saint-Etienne, Bordeaux, Lyon, Toulouse, Caen, and Paris Lariboisière) via the GRIO (Groupe de Recherche et d’Information sur les Ostéoporoses) and the French Rare Bone diseases Network (Filière Maladies Rares OSCAR-Os, Cartilage, Calcium); these centers were contacted between January 2018 and December 2019. A flow-chart summarizes the selection of patients (Fig. 1).

Clinical data were obtained by retrospective chart review. The inclusion criteria were as follows:

- Adult women with previously diagnosed OI following bone experts’ opinion, according to clinical and radiological criteria, namely (1) spontaneous fractures; (2) and skeletal features (wormian bones and/or decreased BMD, scoliosis, or joint hyperlaxity); extra-skeletal features (hearing loss, blue sclera, dentinogenesis imperfecta) supporting OI diagnosis, and exclusion of other causes of bone fragility. Patients with other genetic causes of bone fragility were excluded.
- At least one pregnancy in the last 10 years.

This study was approved by the local Ethical Review Board (local ethics committee for the Cochin Hospital Publications). Written informed consent was obtained from all patients. Only patients with complete data regarding pregnancies (age, number, occurrence of fractures during pregnancy or in the 6 months following delivery, and if so, localization of pregnancy-related fractures, and breastfeeding yes/no) were analyzed. A total of 40 and 23 OI women followed at Cochin hospital and other Departments with bone expertise respectively, were identified as having at least one pregnancy in the last 10 years. Among the 63 women identified, 4 patients were excluded because they actually had other genetic causes of bone fragility (osteoporosis-pseudoglioma, and other rare bone diseases), and 9 were excluded because of insufficient data. Therefore, a total of 50 OI patients with at least one pregnancy were analyzed.

Collected data included: age, weight, height, OI type, molecular diagnosis, OI-related symptoms (i.e. blue sclerae, hearing impairment, scoliosis, joint laxity), number and localization of fractures, history of orthopaedic surgery, bisphosphonates administration and age, other diseases, osteoporosis risk factors other than OI (i.e. smoking, BMI < 19 kg/m^2^, corticosteroids, osteoporosis-inducing disease, low 25-hydroxyvitamin D (25OHD), low calcium intake. Information on pregnancies included: age of pregnancy, number of pregnancies, breastfeeding and if so duration, occurrence of fractures during pregnancy or post-partum and if so date and localization of fracture. Molecular diagnosis, when available, was also collected.

Hip (total hip, femoral neck), or L1–L4 lumbar spine areal bone mineral density (BMD) was available for 42/50 OI patients. We collected the closest BMD to the time of pregnancy. Only Z-score were analyzed since measurements had been performed on different densitometers.

### Statistical analysis

Quantitative data are presented as mean ± standard deviation (SD), and Categorical data are presented as frequencies and percentages (%). Differences between fractured and non-fractured patients were tested for significance using the Mann–Whitney test for quantitative data, and categorical data were compared using the chi-square test and fisher’s exact test when appropriate. Regarding the comparison of pregnancy-related factors (Table 5), and considering that some patients had undergone several pregnancies, and that we had collected this information for each pregnancy, we used as denominator the total number of pregnancies for each group. A *p*-value < 0.05 was considered statistically significant. All statistical analyses were performed using MedCalc software (MedCalc® v11.6.1).

## Results

### General characteristics of OI female patients with pregnancies (Table 1)

The general characteristics of OI patients are detailed in Table 1. The majority of patients (53.1%) had a history of 10 fractures or more before pregnancy, 18.4% had experienced between 5 and 10 fractures, and 30.6% had less than 5 fractures before pregnancy. The mean age at diagnosis was 13.7 ± 14.8 years. In this cohort, 24/37 (64.8%) patients were diagnosed with OI during childhood (8 patients during their 1st year of life, 8 between 1 and 2 years-old, 6 patients between 2 and 10 years-old, and 2 between 10 and 18 years of age. The other 13 patients with available data were diagnosed during adulthood (35.1%). This cohort included 78% of OI type I, 13% of type III, 5% of type IV, and 3% with type V. Molecular diagnosis of OI genes was confirmed in 30/50 (60%) patients: *COL1A1* (70% of molecular diagnoses, *COL1A2* 22%, *IFITM5* 4%, *FKBP10* 4%). No patients had a recessive form of OI. Blue sclera were noted in 84.2% of patients, joint hyperlaxity in 52.6%, and hearing loss in 30.0% of patients. Clinically relevant scoliosis or kyphosis was reported in 51.3% of patients. Severe forms included 21.3% of patients with a history of spine surgery or intramedullary rodding procedures, and 18.6% of patients with walking disability. The mean delay between the last fracture and pregnancy was 10.2 ± 7.7 years. Bisphosphonates had been used in 10/50 (20%) of patients before pregnancy, with a mean delay between last treatment and pregnancy of 8.1 ± 4.9 years. The 50 analyzed patients experienced a total of 83 pregnancies: one pregnancy in 52% of cases, two pregnancies in 34% of cases, 3 pregnancies in 12% of cases, and 5 pregnancy in one (2%) case. The mean age at pregnancy was 29.7 ± 5.1 years-old.

**Table 1:** Characteristics of the female OI cohort with pregnancies

OI female cohort	Total OI cohort 50 patients
Type I, N (%)	28/36 **(77.8)**
Genetically confirmed (%)	30/50 **(60.0)**
Number of fractures before pregnancy < 5, N (%)	15/49 **(30.6)**
Number of fractures before pregnancy 5–10, N (%)	9/49 **(18.4)**
Number of fractures before pregnancy 11–50, N (%)	22/49 **(44.9)**
Number of fractures before pregnancy > 50, N (%)	4/49 **(8.2)**
Age at OI diagnosis (years, mean ± SD)	13.7 ± 14.8
Blue sclera, N (%)	32/38 **(84.2)**
Deafness, N (%)	12/40 **(30.0)**
Joint Hyperlaxity, N (%)	20/38 **(52.6)**
Scoliosis or kyphosis, N (%)	20/39 **(51.3)**
Spine surgery or telescopic rods, N (%)	10/48 **(21.3)**
Wheelchair or walking stick, N (%)	8/46 **(18.6)**
Time between last fracture and pregnancy (years, mean ± SD)	10.2 ± 7.7
Bisphosphonates before pregnancy, N (%)	10/50 **(20.0)**
Time since bisphosphonates (years, mean ± SD)	8.1 ± 4.9
Height (cm, mean ± SD)	153.8 ± 12.7
Weight (kg, mean ± SD)	56.8 ± 12.8
BMI (kg/m^2^, mean ± SD)	24.1 ± 4.9

Bold refers to %

### Characteristics of fracture events during pregnancy and post-partum (Table 2)

Of the 50 patients analyzed, 12 patients (24%) had a history of one fracture or more during pregnancy or in the 6 months following delivery. Clinical characteristics of the 12 OI patients with fractures are detailed in Table 2. Among these, 2 had fractures during two consecutive pregnancies: one patient had a pelvic fracture during her 1st and 2nd pregnancy, and the second had vertebral fractures during her 4th and 5th pregnancies leading to OI diagnosis at 39 years-old. Two other patients experienced fractures both during pregnancy and after delivery. Therefore, we analyzed a total of 16 fracture events (16/83, 19% of OI pregnancies in this cohort). Among the fractured OI patients, half of them experienced a fracture during the 3rd trimester of pregnancy, and the other half during post-partum, with a mean delay of 8 weeks following delivery. None of the patients experienced fractures during childbirth.

**Table 2:** Detailed Characteristics of Osteogenesis Imperfecta women with fractures during pregnancy and post-partum

	P1	P2.1	P2.2	P3	P4	P5	P6	P7.1	P7.2	P8	P9	P10	P11	P12
Age at pregnancy-related Fracture	25	32	34	36	26	36	28	39	41	29	34	32	31	31
Number of pregnancy at fracture	2nd	1st	2nd	1st	1st	1st (twins)	1st	4th	5th	1st	1st	1st	2nd	1st
OI type	NA	I		I	I	I	III	I		I	V	NA	I	I
Molecular diagnosis	NA	COL1A1		COL1A1	COL1A1	NA	NA	COL1A1		COL1A2	IFITM5	COL1A1	NA	COL1
Age at OI diagnosis (years)	2	32		NA	18	1	NA	39		36 years	1st year	1	NA	NA
Height (cm)	164	157		157	153	155	137.5	153.5		151	120	NA	158	164
Weight (kg)	61	48		60	87	42	48	67		NA	40	NA	55	63
BMI	22.7	19.5		24.3	37.1	17.5	25.3	28.4		NA	27.8	NA	22.0	23.4
Blue sclera	Yes	Yes	Yes	Yes	Yes	Yes	Yes	Yes		Yes	Yes	Yes	Yes	NA
Deafness	Yes	No	No	Yes	Yes Transmission hearing loss	No	NA	No		No	No	No	Yes Stapedian prothesis	NA
Joint Hyperlaxity	Yes	Yes		Yes	No	Yes	Yes	No		Yes	No	No	NA	NA
Risk factors for osteoporosis	No	Anorexia 25–28 years old Poor calcium intake until 28 years old		Smoking	No	No	Immobilization and poor calcium intake	No		2 months immobilization 1st pregnancy, smoking before and after pregnancy	1 year immobilization 25 years Smoking stopped at 30 years old	Crohn’s disease	No	AS
Other diseases		Anorexia		Ménière, knee sprain	Hypothyroidism	No	NA	Hypertension				Crohn’s disease	Renal stones	Ankylosing spondylitis B27 + , Adalimumab
Number of fractures in childhood	3	11–50		40	≤ 5	≤ 5	≤ 5	6–10		≤ 5	> 50	11–50	6–10	6–10
Scoliosis	Yes	No	No	No	NA	Yes	No	No		Yes	Yes	NA	Yes	No
Bone densitometry (Z-score)	NA	Spine—2 DS, hip—1.1DS		Spine—2.DS, hip—1.2DS	Spine—0.4 DS, hip—1 DS	Spine—3.3DS, hip—2.6DS	Spine—1.9 DS, hip—1.2DS	Spine—3.7DS, hip—2.7DS		NA	NA	Spine—2.7DS, hip—2.2DS	Spine—3.DS, hip NA	Spine—5.5DS, hip NA
Orthopedic surgery	No	Wrist osteosynthesis		No	No	Shoulder dislocation	NA	No		No	Telescopic intramedullary rod in inferior limbs and Vertebral arthrodesis	No	No	No
Time since last fracture	20 years	4 years		2 years	11 years	5 years	NA	8 years		19 years	17 years	1 year	17 years	5 years
History of bisphosphonates	No	No		No	No	No	Yes Pamidronate 10–12 years old	No		No	Yes Pamidronate 25–26 years old	No	No	No
Time since last bisphosphonates	–	–		–	–	–	Yes 16 years	–		–	Yes 9 years	–	–	–
Term of fracture	Pregnancy 3rd trimester	3rd trimester (8th month)	3rd trimester (8th month)	Pregnancy term unknown	30SA	3rd trimester	34 SA	2 months post partum	Back pain 6 months of pregnancy	1.5 month post partum	1 month post-partum	2 weeks post-partum	6 months post-partum	6 weeks post-partum
Fracture localization	Ankle	Sacrum (left)	Sacrum (right)	Ribs (multiple)	Femoral head, trochanter in post partum	Femoral neck Garden 2	Distal Femur	T10	T6 T7 T12 L1 L5	T8	Wrist	Femoral head	Femoral head	T11 T2 L1
Mechanism of fracture	traumatic	Low trauma (swimming with palms)	Spontaneous	Spontaneous	Spontaneous	Spontaneous	traumatic	Spontaneous	Spontaneous	Low trauma (picking up baby)	Low trauma	Spontaneous	NA	Spontaneous
Breastfeeding	No	Yes	Yes	No	Yes	Yes	Yes	Yes	Yes	Yes	Yes	Yes	Yes	Yes
Breastfeeding duration (months)		1	1		6	4	0.1	18	18	1.5	0.3	0.5	6	1.5

Patients 1 to 12 (P1 to P12). P2 and P7 had fractures for two consecutive pregnancies: P2.1 and P2.2; and P7.1 and P7.2

**Fig. 2:**
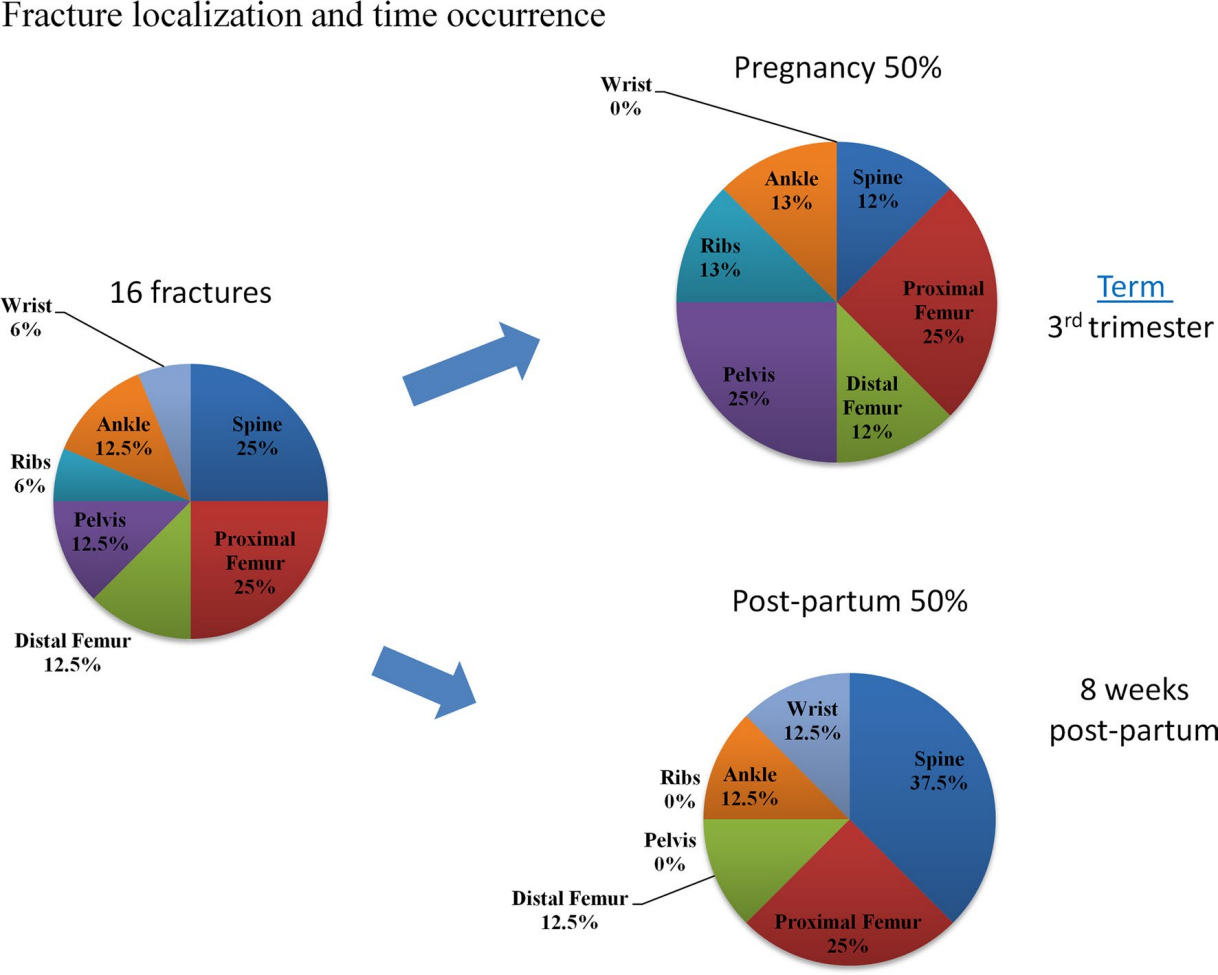
Localization of fractures during pregnancy and post-partum

The most frequent localizations of fractures were: proximal femur (25%), spine (25%), distal femur (12.5%), and pelvis (12.5%) (Fig. 2). During pregnancy, the most frequent localizations were proximal femur (25%) and pelvis (25%). During post-partum, the most frequent fracture localizations were the spine (37%) and proximal femur (25%). Fractures occurred either spontaneously, after a low trauma or a significant trauma in 69%, 19% and 12% of cases, respectively. Detailed localizations of pregnancy and post-partum-related fractures in the OI cohort are reported in Table 2.

### Clinical characteristics comparison of fractured and non-fractured OI patients (Tables 3, 4, 5)

We then compared the group of 12 OI women with fractures during pregnancy or within 6 months post-partum, with the 38 OI women without a history of fracture in the same period. We analysed three different types of parameters: (1) OI related characteristics (Table 3), (2) osteoporosis risk factors other than OI (Table 4), and (3) pregnancy and breastfeeding-related factors (Table 5). The mean delay between pregnancy and BMD was + 5.4 ± 50.1 months.

**Table 3:** Comparison of fractured and non-fractured patients

OI female cohort 50 patients	Patients with Pregnancy-related Fractures* 12 patients	Patients with no fracture 38 patients	P*
Type I, N (%)	10/12 **(83.3)**	18/24 **(75.0)**	0.69
Genetically confirmed (%)	8/12 **(66.7)**	22/38 **(57.9)**	0.74
Age at OI diagnosis (years, mean ± SD)	18.4 ± 17.3	12.6 ± 14.3	0.24
Diagnosis in childhood, N (%)	4/9 (44.4)	19/27 (70.4)	0.23
Number of fractures before pregnancy < 5, N (%)	5/11 **(45.5)**	10/38 **(26.3)**	0.275
Number of fractures before pregnancy 5–10, N (%)	2/11 **(18.2)**	7/38 **(18.4)**	1.0
Number of fractures before pregnancy 11–50, N (%)	3/11 **(27.3)**	19/38 **(50.0)**	0.30
Number of fractures before pregnancy > 50, N (%)	1/11 **(9.1)**	3/38 **(7.9)**	1.0
Blue sclera, N (%)	11/11 **(100)**	21/27 **(77.8)**	0.15
Deafness, N (%)	4/12 **(36.4)**	8/30 **(26.7)**	0.72
Joint Hyperlaxity, N (%)	6/10 **(60.0)**	14/28 **(50.0)**	0.72
Scoliosis or kyphosis, N (%)	5/10 **(50.0)**	16/29 **(55.2)**	1.0
Spine surgery or telescopic rods, N (%)	1/11 **(9.1)**	9/37 **(25.7)**	0.42
Wheelchair or walking stick, N (%)	2/11 **(18.2)**	6/32 **(18.75)**	1.0
Time between last fracture and pregnancy (years, mean ± SD)	10.5 ± 8.1	10.1 ± 7.3	0.94
Bisphosphonates before pregnancy, N (%)	2/12 **(16.7)**	8/38 **(21.1)**	1.0
Time since bisphosphonates (years, mean ± SD)	12.5 ± 4.9	7.0 ± 4.5	0.18
Height (cm, mean ± SD)	154.8 ± 10.9	153.4 ± 13.5	0.35
Weight (kg, mean ± SD)	58.2 ± 12.2	56.6 ± 12.6	0.98
BMI (kg/m^2^, mean ± SD)	24.8 ± 5.5	23.9 ± 4.7	0.52

* % pregnancy-related fractures (during pregnancy or within 6 months after delivery); % and statistics were calculated on the basis of available data; a *p*-value < 0.05 was considered significant. Bold refers to %

**Table 4:** Comparison of Low bone mass risk factors in fractured and non-fractured OI patients

	Fractures N = 12	No fracture N = 38	*p*
Smoking (%)	2/12 (16.7)	10/29 (34.5)	0.45
BMI < 19 kg/m2 (%)	2/10 (20)	2/29 (6.9)	0.27
Corticosteroids (%)	0	1/29 (3.4)	1
*Osteoporosis inducing disease*			
Spondyloarthritis	1/12 (8.3)	0	0.2
Inflammatory bowel disease	1/12 (8.3)	0	0.2
Anorexia	1/12 (8.3)	1/29 (3.4)	0.50
Rheumatoid arthritis	0	1/29 (3.4)	1.0
Immobilization	3/10 (30.0)	3/29 (10.3)	0.16
25OHD < 30 ng/ml (%)	2/10 (20.0)	13/27 (48.1)	0.15
Low calcium intake (%)	1/12 (8.3)	1/29 (3.4)	0.50
Spine BMD (Z-score)	− 2.93 ± 1.57	− 1.48 ± 1.67	0.03
Femoral neck BMD (Z-score)	− 2.25 ± 0.90	− 0.7 ± 1.19	0.09
Total hip BMD (Z-score)	− 2.05 ± 0.74	− 0.53 ± 1.36	0.04
At least 1 risk factor, %	9/11 (81.8)	17/29 (58.6)	0.27

25OHD: 25-hydroxyvitamin D; BMD: Bone mineral density;

**Table 5:** Comparison of Pregnancy and lactation-related factors in fractured and non-fractured OI patients

	Fractures N = 12	No fracture N = 38	*p*
Age (years; mean ± SD)	32.7 ± 3.1	29.3 ± 5.0	0.002
Number of pregnancies (mean ± SD)	0.5 ± 0.9	0.6 ± 0.8	0.6
Time since last pregnancy (years, mean ± SD)	4.1 ± 2.7	4.4 ± 2.0	0.51
Duration of lactation after previous pregnancy (months, mean ± SD)	5.5 ± 8.6	2.55 ± 4.8	0.86
Breastfeeding (yes, %)*	12/14 (85.7%) Including 100% of patients with post-partum fractures	25/53 (47.1%)	0.03

* For pregnancy-related factors, data presented are the number of occurrence of each variable (e.g. breastfeeding) on the total number of pregnancies in each group

No differences were observed regarding the OI phenotype, including the number of fractures in childhood, history of scoliosis, spine or long bone surgery (Table 3). Bisphosphonates had been administered before pregnancy in 16.7% of fractured patients (pamidronate n = 2) vs 21.1% of non-fractured patients (pamidronate n = 3, zoledronate n = 5, alendronate n = 1, some patients had received both pamidronate and zoledronate). The mean delay since bisphosphonates was 12.5 ± 4.9 years in the fractured group versus 7.0 ± 4.5 years in the non-fractured group (not significant). The minimum delay between last bisphosphonates and pregnancy was 1 year, no patients had received bisphosphonates during pregnancy.

The comparison of classic osteoporosis risk factors showed that the fractured OI group had lower BMD at the spine (Z-score − 2.9 ± 1.6DS versus − 1.5 ± 1.7DS, *p* = 0.03), and at the total hip (− 2.0 ± 0.7DS versus − 0.5 ± 1.4DS, *p* = 0.04) than the non-fractured group (Table 4). At least one risk factor for osteoporosis other than OI was found in 81.8% of the fractured group vs 58.6% of the non-fractured group, but this difference was not significant.

Regarding pregnancy-related factors, we observed a striking difference in breastfeeding rate (Table 5). Indeed, breastfeeding was reported in 85.7% of the fractured group, vs 47.1% of the non-fractured group (*p* = 0.03). Furthermore, post-partum fractures only occurred in breastfeeding patients. More specifically, the frequency of breastfeeding was 8/8 (100%) in post-partum fractures, vs 25/53 (47.1%) of post-partum without fractures (*p* = 0.006). We also observed that mean age at pregnancy was slightly higher in the fractured group (32.7 ± 3.1 years, versus 29.3 ± 5.0 years, *p* = 0.002).

## Discussion

Our study shows that beyond the increased fracture risk due to their underlying genetic disease, OI patients have also an increased risk of fracture during pregnancy. Moreover, our study suggests that breastfeeding may be a strong risk factor for fracture in these patients.

To our knowledge, this is the first study with the aim of describing the characteristics of fractures occurring during pregnancy and post-partum in patients with OI. So far, only limited and generally self-reported data were available [15, 16]. While McAllion et al.reported 4.2% of vertebral crush fractures in a series of 100 pregnant OI patients, only one stress fracture was reported in a retrospective cohort of 295 pregnant women with OI [17]. Furthermore, no post-partum data are currently available in OI patients.

In women without bone fragility disease, comparison of BMD measurements before and after pregnancy shows BMD decrease during pregnancy [10, 18]. While moderate BMD declines are described during pregnancy (− 1.8% at the spine, and − 3.2% at the total hip during pregnancy)[10], even more important declines are reported during lactation, as shown by progressive 5–10% lumbar spine aBMD decline during the first 3–6 months of lactation [13, 19], and up to 10–15% loss of trabecular aBMD in lactating adolescents [20, 21]. The initial rapid bone loss is followed by a subsequent recovery of bone mineral with weaning and with recovery of menses [13, 19].

In the present OI cohort, 24% of patients experienced at least one pregnancy or post-partum fracture. This suggests a dramatically higher proportion of fractures as compared with the very rare observations of fractures during or following pregnancy in the general female population, described under the term “pregnancy and lactation associated osteoporosis” [22]. In a French retrospective cohort study of 52 women with pregnancy-related fractures, 2 out of 52 patients had a diagnosis of OI revealed by pregnancy fractures [14]. The diagnosis of OI was also made in 1/10 (10%) of fractured patients in the British cohort described by Hardcastle et al. [23], suggesting that genetic factors may contribute to pregnancy and lactation-related osteoporosis, and suggest the relevance of the assessment of an underlying genetic disease in adult female patients with fractures of unknown origin during pregnancy or during post-partum. This also suggests that pregnancy and breastfeeding may contribute to decompensating bone fragility in patients with OI.

In comparison with pregnancy-related fracture cohorts, this OI female cohort displayed fractures not only at the spine, which represent 25% of all fractures, and 37% of post-partum fractures, but also femoral fractures representing more than 30% of all fractures when proximal and distal femoral fractures were combined. These femoral fractures were not diaphyseal fractures on telescopic rods as may have been expected in severe OI forms, but mostly femoral neck fractures, and subchondral fractures of the femoral head. The present OI female cohort also presents similarities with the cohorts with pregnancy-related osteoporosis: fractures occurred mainly during the 3rd trimester or within 2 months post-partum. Most patients had an osteoporosis-inducing disease or risk factor other than OI before pregnancy (81.8% in the present study, versus 63% in the French cohort). Recurrence of fractures during consecutive pregnancies was rare: 2 out of 12 patients (17.7%) vs 19.2% in the cohort by Laroche et al. [14].

Importantly, this study identified an association between breastfeeding and post-partum fractures, as all fractures in the post-partum occurred in breastfeeding women. This suggests a deleterious effect of breastfeeding on bone fragility fractures in the context of OI. Interestingly, breastfeeding had been contra-indicated before pregnancy in all patients followed in Cochin Hospital but non-compliance of this recommendation by patients was frequent. Physicians should therefore inform female patients of this risk.

Patients included in this cohort had a mild to moderate OI phenotype, as shown by a vast majority of type I OI, a mean height only slightly below normal, a low prevalence of spine and long bone surgery and of mobility assistance. The rate of type I OI in our cohort is consistent with those reported by Yimyang et al. (78.8% of OI type I) and McAllion et al. (86.8%) when describing obstetrical outcomes in OI [15, 16]. These observations certainly reflect the reality of pregnancy in OI women, with a limited number of pregnancies in more severe forms.

Study limitations included the limited number of fractures. Second, the retrospective design of this study did not provide information on the exact age of onset which may be different from the age of diagnosis with some patients describing their first fracture sometimes years before the diagnosis of OI. This may have minimized the comparisons of fractured vs non fractured patients regarding age of onset. In this retrospective study, some data were not available for all patients. We did not have data on BMD levels before and after pregnancy in this OI cohort, which would contribute to the understanding of BMD evolution during and after lactation in this context. In addition, we were unable to have detail on weight variation during pregnancies. Finally, this cohort may not be representative of all OI pregnancies since all patients were taken care of in tertiary Centers, with thorough medical follow-up by specialists in Rare Bone disorders. Based on our data, a more specific and thorough follow-up of these patients should be proposed and endorsed by reference centers in the future.

This study also had several strengths. Although OI is a rare disease, we were able to examine a relatively large group of patients. We managed to collect extensive clinical data on OI phenotype, fracture events during and following pregnancy, localization of fracture, osteoporosis risk factors, previous treatments, and BMD.

In conclusion, we reported the largest OI female cohort with pregnancy and breastfeeding- associated fractures. Despite the mild OI phenotype of patients included in this cohort, 16 fractures, mainly vertebral or femoral, were observed in a total of 83 pregnancies. Most fractures occurred at the end of pregnancy or during the first months of post-partum, in patients with at least one low bone mass risk factor other than OI, or low BMD. Breastfeeding appears to be deleterious as all post-partum fractures occurred in breastfeeding patients and should therefore be avoided. These results highlight the importance of anticipating pregnancies in OI patients, with a tight control of modifiable risk factors during pregnancy and avoiding breastfeeding.

## Data Availability

The data that support the findings of this study are available from the corresponding author upon reasonable request.
